# Practical Notes and Formulæ

**Published:** 1886-07-20

**Authors:** 


### PRACTICAL NOTES AND FORMULÆ.

Dr. Charles N. Dixon Jones, Surgeon to Woman's Hospital, Brooklyn, and Instructor to New York Polyclinic, writes: I think that I have found another use for cocaine. The applications for this drug are so numerous and its therapeutic uses so various that it is very difficult to say just what is new. Gastric lavage is rapidly becoming our most reliable treatment in chronic dyspepsia and gastritis. In order to avoid some of the disagreeable features attending this operation I have resorted to the following expedient: About fifteen minutes before commencing the operation, the patient is allowed to hold in his month a piece of absorbent cotton saturated with a 4 per cent. solution of hydrochlorate of cocaine. In a few minutes the palate and fauces are painted with the same solution. The stomach tube is lubricated with a mixture of olive oil, oil of wintergreen and cocaine, after which the tube may be introduced and the stomach irrigated without that disagreeable vomiting of the tube and efforts at gaging which frequently attend the operation. I have adopted this method in some of the most trying and difficult cases, and always with the most happy result.

**For Cholera Morbus and Dysentery of Children.**.—Dr. E. Van Goidtsnoven writes that he has used with excellent results in the bowel affections of the children the following prescriptions:

℞. Water....................................℥ iv,
Chloranodyne (P. D. & Co.)......................gtt. x,
Sulph. morphine..............................gr. $\frac{1}{8}$.

M. Dose teaspoonful every three hours to a child two to four years old. In very young children omit the morphine as there is a portion already in the chloranodyne. In conjunction with the above use the following:

℞. Bichloride mercury..........................gr. $\frac{1}{4}$,

Water....................................℥ iv.

M. Teaspoonful every three hours, alternating with the above until the color of the evacuations change or assume a better appearance. In addition to this the bowels are washed out twice a day with a pint and a half of water, containing a teaspoonful of the salicylate of soda; or in lieu of this a teaspoonful of common salt may be used. This treatment, in appropriate doses, is also excellent in adult cases.

**Dysentery.**—The following is Deliouz's formula, or the Brazilian method for teating which claims great success:

℞. Powdered ipecac.............................ʒ j,  
Boil five minutes in water......................℥ x,  
Filter and add tinc. opii........................ʒ j,  
Syrup ginger.......................................ʒ viiss,  
Peppermint water..................................℥ j.  
M. Take a tablespoonful every hour. The opiate and the flavoring is to guard against vomiting, and in some instances the dose will require reduction to prevent undue nausea. The remedy must be continued until bilious discharges ensue, when it should be supended, or continued in reduced quantity. In the meanwhile mustard should be applied to the abdomen followed by emollient poultices and rest in bed.

**For Impotence.**—Dr. Bartholow highly recommends the following pill, in impotence:

℞. Ext. cannabis ind...............................gr. x,  
Ext. ergot aq........................................℈ ij,  
Ext. nucis vom......................................gr. x.  
M. Ft. pil. xx. Sig. One, night and morning.—*American Medical Journal.*

**Flatulent Dyspepsia.**—Professor Parvin frequently prescribes

℞. Alomi..................................................gr. j,  
Resinæ podophyllin................................gr. iss,  
Ext. nucis vomicæ.................................grs. iij,  
Ext. belladonnæ....................................gr. j.  
M. Ft. pilulæ No. xii. Sig. One pill after meals.—*New Eng. Med. Monthly.*

**Malarial Hematuɹia.**—Dr. T. J. Draper recomends—

℞. Tinct. nucis vomicæ..............................*m* xxx,  
Tinct. iodi................................................*m* xxx,  
Tinct. hyoscami.........................................ʒ j,  
Ext. buchu, fl.............................................℥ j,  
Sat. sol. sodii sulphatis..............................℥ ij.  
M. Sig. Teaspoonful every two hours.—*Weekly Medical Review.*

**Treatment of Acute Tonsillitis.**—Dr. John Brown states, in the *British Medical Journal*, that it is a rare event for suppuration to occur in acute tonsillitis, if treated early with following mixture:

℞. Sodii salicylat.......................................ʒ iss,  
Pot. bicarb................................................ʒ iss,  
Tinct. aconit...............................................*m* 40,  
Liq. opii sed...............................................*m* 30,  
Sp. chloroform.............................................ʒ ii,  
Aq. ad.......................................................℥ viii.  
M. One ounce to be taken every two or three hours for the first thirty-six hours. The same mixture is his sheet-anchor for

